# p38 MAPK activation and H3K4 trimethylation is decreased by lactate *in vitro* and high intensity resistance training in human skeletal muscle

**DOI:** 10.1371/journal.pone.0176609

**Published:** 2017-05-03

**Authors:** Lena Willkomm, Sebastian Gehlert, Daniel Jacko, Thorsten Schiffer, Wilhelm Bloch

**Affiliations:** 1 Department of Molecular and Cellular Sports Medicine, Institute of Cardiovascular Research and Sports Medicine, German Sport University Cologne, Cologne, Germany; 2 Outpatient Clinic for Sports Traumatology, German Sport University Cologne, Cologne, Germany; University of Birmingham, UNITED KINGDOM

## Abstract

Exercise induces adaptation of skeletal muscle by acutely modulating intracellular signaling, gene expression, protein turnover and myogenic activation of skeletal muscle stem cells (Satellite cells, SCs). Lactate (La)-induced metabolic stimulation alone has been shown to modify SC proliferation and differentiation. Although the mechanistic basis remains elusive, it was demonstrated that La affects signaling via p38 mitogen activated protein kinase (p38 MAPK) which might contribute to trimethylation of histone 3 lysine 4 (H3K4me3) known to regulate satellite cell proliferation and differentiation. We investigated the effects of La on p38 MAPK and H3K4me3 in a model of activated SCs. Differentiating C2C12 myoblasts were treated with La (20 mM) and samples analysed using qRT-PCR, immunofluorescence, and western blotting. We determined a reduction of p38 MAPK phosphorylation, decreased H3K4me3 and reduced expression of Myf5, myogenin, and myosin heavy chain (MHC) leading to decreased differentiation of La-treated C2C12 cells after 5 days of repeated La treatment. We further investigated whether this regulatory pathway would be affected in human skeletal muscle by the application of two different resistance exercise regimes (RE) associated with distinct metabolic demands and blood La accumulation. Muscle biopsies were obtained 15, 30 min, 1, 4, and 24 h post exercise after moderate intensity RE (STD) vs. high intensity RE (HIT). Consistent with *in vitro* results, reduced p38 phosphorylation and blunted H3K4me3 were also observed upon metabolically demanding HIT RE in human skeletal muscle. Our data provide evidence that La-accumulation acutely affects p38 MAPK signaling, gene expression and thereby cell differentiation and adaptation *in vitro*, and likely *in vivo*.

## Introduction

Single bouts of exercise activate molecular signaling pathways in skeletal muscle tissue modifying gene expression and protein turnover to adapt to increased demand [1]. Although there is considerable knowledge about the molecular regulation of these mechanisms, the specific role of metabolic stimulation (e.g. lactate accumulation) remains elusive. Increased lactate (La) concentrations [2, 3], as observed in working muscle, impact intracellular signaling in myoblasts [4], and myotubes [5] and can be considered as a metabolic modifier of molecular skeletal muscle adaptation.

Mitogen activated protein kinase p38 (p38 MAPK) is an important regulator of activation, proliferation and differentiation of skeletal muscle stem cells (SCs) during skeletal muscle myogenesis [6, 7]. p38 MAPK is phosphorylated (p-p38) and activated by the upstream MAPK kinases 3 and 6 (MKK3 and MKK6) at threonine 180 (T180) and tyrosine 182 (Y182) and directs switch/sucrose non-fermentable (SWI/SNF) and Ash2L-KMT containing complexes to regulatory muscle genes, promoting their transcription [8–10]. Furthermore, p38 is directed to the chromatin of muscle specific genes through interactions with E47 [11], Mef2d [12] and BAF60 [10] and inducing their transcription. CAM-downregulated by oncogenes (CDO) is a transmembrane protein of the Ig-fibronectin III repeat subfamily. It can activate p38 MAPK which in turn leads to the formation of MyoD-E47 heterodimers that bind to E-box sequences on the regulatory regions of muscle-specific genes [13, 14]. Additionally, H3K4me3 has been shown to play a major role during myogenesis being essential in skeletal muscle regeneration [15] and hypertrophy [16, 17]. Its trimethylation via a Pax7-dependent recruitment of a histone methyl transferase (HMT) initiates Myf5 expression in proliferating myoblasts [18]. Furthermore, Rampalli et al. [12] showed that Ash2L is recruited by Mef2d and targets the H3K4me3 mark to myogenin promoters and that this recruitment is regulated by p38 MAPK phosphorylation of Mef2d, directly linking the p38 MAPK signaling pathway to H3K4e3 [12, 19]. This suggests an important role of p-p38 MAPK-H3K4me3 by modulating the expression of myogenic factors that regulate satellite cell differentiation and potentially skeletal muscle adaptation.

However, it remains to be determined whether La can effect p38 MAPK signaling, h3K4me3 and muscle-specific gene expression in a model of activated SCs (C2C12 myoblasts) and whether this also occurs in differentiated adult human skeletal muscle. To establish a role for La as a metabolic signal for gene expression [5, 20], we firstly investigated whether elevated La levels—as observed after high intensity exercise within human skeletal muscle [21–24] are able to influence p38 MAPK phosphorylation, H3K4me3 and subsequent gene expression in differentiating C2C12 cells. Secondly, we investigated whether this signaling pathway remains sensitive to acute exercise-induced metabolic stimulation in differentiated human skeletal muscle tissue. To simulate acute increases in metabolic demands of skeletal muscle and La production we stimulated human skeletal muscle with acute resistance exercise protocols with moderate (STD) or very high intensity (HIT) muscle contractions and investigated p-p38 and H3K4me3 signaling in post exercise skeletal muscle biopsies. We determined that La-treatment of C2C12 cells and high intensity muscle contractions with increased La-levels, transiently decrease p-p38 and H3K4me3 signaling associated with a temporal reduction in myogenic responses.

## Methods

### Cell culture and treatment

C2C12 mouse myoblasts (DSZM Braunschweig, Germany) were kept in cell culture flasks (BD Falcon, Bedford, USA) at 37°C and 5% CO_2_ in proliferation medium (PM; DMEM, 1% penicillin-streptomycin, 4 mM Glutamine, 1.5 g/L sodium bicarbonate, 1 mM sodium pyruvate (all from Invitrogen, Karlsruhe, Germany), 20% fetal calf serum (PAA, Pasching, Austria). For experimental procedures, myoblasts were seeded on Petri dishes or gelatin-coated (0.1% in PBS) glass cover slips at a density of 10.000 cells per cm2. After plating, myoblasts were kept in PM for 48 h. For experimental purposes, PM was switched to differentiation medium (DM; DMEM, 1% penicillin/streptomycin, 4 mM glutamine, 1.5 g/L sodium bicarbonate, 1 mM sodium pyruvate, and 4% horse serum (all from Invitrogen, Karlsruhe, Germany). Control samples were treated with La-free, i.e. 0 mM La DM. Cells receiving the La intervention were incubated with 20 mM La DM (L-sodium lactate; Sigma-Aldrich, Steinheim, Germany). Similar and higher La-concentrations were observed in human blood and muscle after vigorous exercise (see Discussion) and were used previously in the literature [5]. In order to investigate acute effects, myoblasts were treated for 15 min, 30 min, 1 h, 4 h, and 24 h with DM and subsequently lysed for Western blot (WB) analysis. Additionally, myoblasts were fixed 15 min, 1 h, and 4 h after the start of treatment followed by immunofluorescent staining (IF). To analyze changes in gene expression, myoblasts were treated with 0 mM (control) or 20 mM La DM for 2 h. Subsequently La was removed followed by incubation with 0 mM La DM for both conditions for 4 h in order to change and detect gene expression exerted from the distinct lactate stimulation patterns. The protocol was applied in this way in order not to expose the cells to the stimulus for longer than in a realistic training situation. At 0 h and 6 h RNA was isolated as described below. To determine whether long-term La effects are regulated by p38 inhibition, myoblasts were treated with 0 mM La DM, 0 mM La DM + DMSO, 0 mM La + 10 μM SB203580, or 20 mM La DM for 2 h each day for 5 days to simulate repeated exercise in a microcycle e.g. Between repeated bouts of incubation all cells were kept in 0 mM La DM. SB203580 is a specific p38 MAPK inhibitor [25], insoluble in water and therefore dissolved in DMSO. To exclude effects being induced by the solvent, 0 mM + DMSO was added as an additional control condition. After the last treatment, cells were fixed for IF or lysed for WB. All *in vitro* experiments were carried out in triplicates.

### Human study

For detailed information on the study, see [26]. Briefly, 15 healthy male subjects participated in the study (age: 23 ± 3 years; height: 180 ± 6 cm; body mass: 76.2 ± 8.3 kg). Participants were informed in oral and written form of the study’s purpose before giving written informed consent. The study was approved by the Ethic Committee of the German Sport University Cologne in compliance with the Declaration of Helsinki. Subjects performed either a STD (n = 7) or a HIT (n = 8) RE protocol.

### Experimental design and strength training protocols

On the evening before reporting to the lab (10 pm), subjects consumed a standardized Fresubin^®^ protein-energy drink (Fresenius Kabi, Bad Homburg, Germany; containing 20 g protein, 24.8 g carbohydrate 13.4 g fat, providing 1260 kJ) and then fasted overnight. The following morning, subjects had a second and equal protein-energy drink (60 min before exercise) to ensure the intervention was carried out in the fed state. One week before the actual trial, participants underwent maximal strength testing on an ISOMED 2000 isokinetic machine (D&R Ferstl GmbH, Hemau, Germany). The highest maximum force achieved was applied for the experimental trial as the 100% reference curve. On the day of the actual experimental trial, subjects reported to the laboratory at 7.45 am. All exercise regimens were carried out as unilateral single-leg extensions with 70° range of movement (ROM).

- STD: 3 sets of 10 unilateral concentric and eccentric leg extensions with 3 min rest between sets. 75% of maximum eccentric and concentric force. The movement speed was determined as 65°·s^-1^ with a loading time of 66 s time under tension (TuT).
- HIT: 1 set of 20 subsequent unilateral concentric and eccentric leg extensions without rest. 100% of the highest possible maximum concentric and eccentric force from the first to the last repetition. Movement speed was determined as 40°·s^-1^ with a loading time of 70 s time under tension (TuT).

Capillary blood was taken before (pre) and at 0, 2, 4, 6, 8, and 10 min after (post) exercise from the earlobe to determine peak La levels. La analysis was performed on a Biosen S-Line analyzer (EKF-Diagnostic GmbH, Barleben, Germany). At 15, 30 min, 1, 4, and 24 h post exercise muscle biopsies were taken from the Musculus vastus lateralis. Muscle tissue samples for western blot analysis were treated as described in [26].

### Gene expression analysis

After treatment, cells were washed with PBS and lysed directly in the culture dish with TriReagent^®^ (1 mL per 106 cells). RNA was subsequently isolated using bromochloropropane (both Molecular Research Center, Cincinnati, OH, USA) according to the manufacturer’s instructions. After the last centrifugation step, supernatants were completely removed, the pellets air-dried and dissolved in RNAse free water (Sigma-Aldrich, Steinheim, Germany). The QuantiTect^®^ Reverse Transcription kit was used for synthesis of cDNA. Procedures were carried out according to the provided instructions. Quantitative real-time RT-PCR was subsequently performed using the QuantiTect^®^ SYBR Green PCR Kit (both from Qiagen, Hilden, Germany) and the Mx3005P (Agilent Technologies, Santa Clara, CA, USA). Primer sequences: *Pax7 Nm_011039)*: forward 5'GCTACAGTGTGGACCCTGTG3' and reverse 5'GAGACTCAGGGCTTGGGAAG3'; *Myf5 (Nm_008656)*: forward 5'TGGTCCCGAAAGAACAGCAG3' and reverse 5'AAGCTGTGTCCTGAAGAGCC3'; *myogenin (Nm_031189)*: forward 5'GTGAATGCAACTCCCACAGC3' and reverse 5'GTTGGGCATGGTTTCGTCTG3'; *myosin heavy chain 1 (MHC1; Nm_30679)*: forward 5'CCAGGACCTTGTGGACAAAC3' and reverse 5'TGGTCACTTTCCTGCACTTG3'; *MHC2 (Nm_00139545*): forward 5'AAGAGACAAGCTGAGGAGGC3' and reverse 'GAATCACACAGGCGCATGAC3'; 174 *cyclophilin A (Nm_203430)*: forward 5'GGATTCATGTGCCAGGGTGG3' and reverse 5'CACATGCTTGCCATCCAGCC3'. The cycling conditions were applied as instructed by the manufacturer for 40 cycles. Samples were run in triplicates. Data were normalized to housekeeping gene (cyclophilin A). Cyclophilin A expression did not change with the intervention (data not shown). The x-fold increase was calculated according to [27].

### Immunofluorescence (IF) of cells

Cells were fixed by incubation with 4% PFA in DPBS for 10 min at the before mentioned time points. Cells were stained as described in [4]. Briefly, cells were permeabilized with a 0.5 M ammonium chloride solution in PBS containing 0.25% Triton X-100. After washing, unspecific binding sites were blocked with 5% BSA in PBS for 1 h at room temperature. Cells were then incubated with primary antibodies H3K4me3 (rabbit; dilution 1:1000; Cell Signaling Technology, Danvers, MA, USA), Pax7 (mouse; dilution 1:250; Neuromics, Edina, NM, USA), Myf5 (rabbit; 1:250; Genetex, Irvine, CA, USA, F5D (myogenin; mouse; dilution 1:250), or Mf20 (myosin heavy chain; mouse; dilution 1:500; both Developmental Studies Hybridoma Bank, Iowa City, IO, USA) in 0.8% BSA in PBS at 4°C overnight. The following day, the primary antibody solutions were washed off and cells incubated with a secondary antibody solution containing Alexa488-conjugated goat-anti-mouse IgG or Alexa488-conjugated goat-anti-rabbit IgG (dilution 1:1000; both from Invitrogen, Karlsruhe, Germany) for 1 h at room temperature. This solution was again washed off and 5 min incubation with DAPI (Sigma-Aldrich, Steinheim, Germany) followed before cells were washed for the last time and cover slips mounted onto microscopic slides using Aqua- Poly/Mount (Polysciences, Warrington, PA, USA).

### Muscle biopsy IF

Muscle biopsy sections were fixed by 8 min incubation with ice-cold acetone at -80°C followed by thorough washing with TBS. After blocking unspecific binding sites with 5% BSA in TBS, cells were incubated with the primary antibodies H3K4me3 (see above) and Dystrophin (MANEX1A-4C7; mouse; dilution 1:75; Developmental Studies Hybridoma Bank, Iowa City, IA, USA) in 0.8% BSA in TBS overnight at 4°C. The procedures on the following day were as described in the cell IF section, except that TBS was used instead of PBS.

### Microscopy

Cells were photographed on an Axiovert 200M microscope (Carl Zeiss, Jena, Germany) equipped with an AxioCam MRm (Carl Zeiss, Jena, Germany). For acute effects on H3K4me3 a 63x objective was used and 20 pictures were taken per condition. All photos were taken with identical settings to allow for follow-up fluorescent semi-quantification. Pictures were analysed using Metamorph 7.0 software (Molecular Devices, Sunnyvale, CA, USA). For this, pictures were adjusted and corrected for background and a common threshold was set to neglect unspecific signals. In case of long-term intermittently incubated C2C12 cells samples were photographed with a 20x objective. 20 pictures were taken per condition. ImageJ 1.46r software (National Institute of Health, Bethesda, MD, USA) was used to automatically count DAPI-positive nuclei. Nuclei positive for Pax7, Myf5, and myogenin as well as nuclei lying within MHC-positive myotubes were counted manually. Human muscle biopsies were photographed using 10x and 63x objectives with identical settings to allow for follow-up semi-quantification. Fluorescent signals were quantified from 10x pictures using the particle analysis tool from ImageJ to calculate the average area covered by H3K4me3 signal within the cell nuclei in the photograph. For detailed pictures using the 63x objective, the ApoTome (Carl Zeiss, Jena, Germany) was additionally utilized to achieve higher image quality.

### Western blot analysis

Cells and muscle biopsies were lysed and blotted as described previously [4, 26]. Cell lysate and pooled muscle homogenate total protein concentrations were determined using the *RC DC*^™^ Protein Assay Kit (Bio-Rad Laboratories, Inc., Hercules, CA, USA). 2x Laemmli buffer (4% sodium dodecyl sulphate, 10% β-mercaptoethanol, 20% glycerol, 0,004% bromphenol blue, 0,125M Tris HCl) was added and incubated for 5 minutes at 95°C. Proteins were separated with gel electrophoresis and afterwards blotted on to a polyvinylidene transfer membrane (Pall Corporation, Port Washington, NY, USA) using the TransBlot Turbo (Bio- Rad Laboratories, Inc., Hercules, CA, USA). Membranes were blocked using 5% BSA or 5% dry-milk in TBST before incubation with the primary antibody (for H3K4me3 see IF section; total H3, rabbit, 1:1000, Cell Signaling Technology, Danvers, MA, USA, phospho-p38 T180 + Y182, rabbit, 1:1000, Epitomics, Burlingame, CA, USA; total p38, rabbit, 1:200, Abcam, Cambridge, UK), actin (mouse, 1:4000; Millipore, Billerica, MA, USA), or tubulin (mouse, 1:2500; Genetex, Irvine, CA, USA) diluted in TBST at 4°C overnight. After washing membranes were incubated with the respective HRP-conjugated secondary antibody (1:2000 in TBST; goat anti-mouse or goat-anti-rabbit; Thermo Fisher Scientific, Waltham, MA, USA). Signals were detected using enhanced chemo-luminescence assay (Amersham Life Science, Buckinghamshire, UK) exposed on Kodak X-OMAT x-ray films (Eastman Kodak Co, Rochester, NY, USA). All blots were not stripped any time. Bands were analyzed using the ImageJ software and normalized to actin or tubulin bands.

### Statistical analysis

All data are presented as mean ± standard deviation (SD) Gene expression and IF data were analyzed for normal distribution using Kolmogorov-Smirnov- or Shapiro-Wilk-tests. Repeated measures analysis of variance was used to test differences over the time course of the experiments. If significance was indicated, a Bonferroni post-hoc test was applied to test for differences between the groups. 2-way analysis of variance for repeated measurements was used to test differences between groups. Afterwards, Holm-Sidak´s correction for multiple comparisons was applied with multiplicity adjusted p-value determination. Statistical analysis was carried out using IBM SPSS Statistics Version 20 for Windows (IBM SPSS Corporation, Chicago, IL, USA). Statistical significance was set at p < 0.05.

## Results

**Fig 1:**
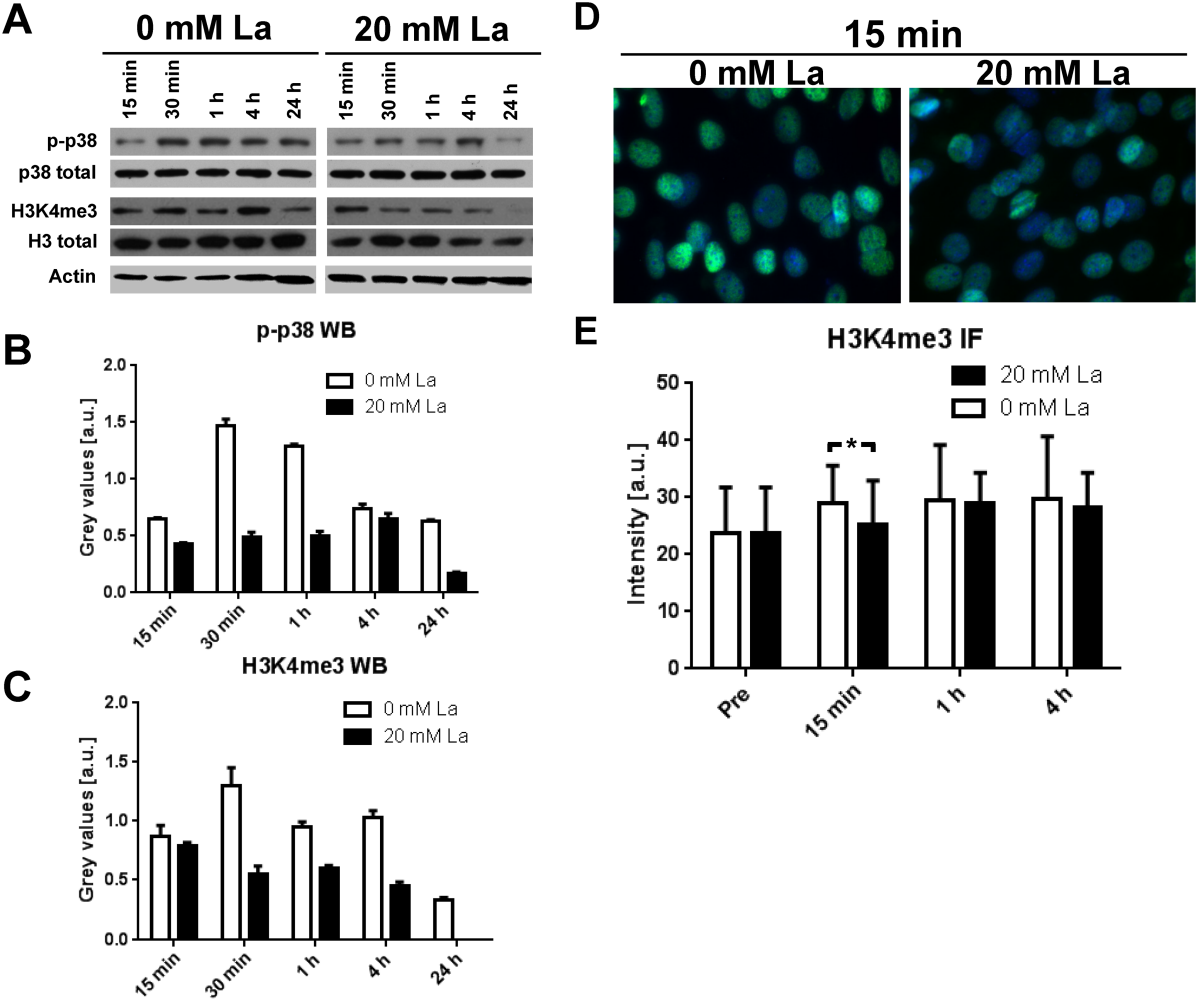
Acute effects of 20 mM La DM incubation on p38 MAPK signaling in differentiating C2C12 myoblasts. **A** Representative western blots of C2C12 cell lysates after up to 24 h of incubation with 0 or 20 mM La DM. **B** Quantification of WBs for p-p38 MAPK at T180 and Y182 (p-p38) revealed increased p-p38 following 0 mM La DM treatment for up to 4 h but unchanged levels in 20 mM La DM condition. **C** Quantification of H3K4 in the trimethylated state (H3K4me3) showed a pattern similar to p-p38 with a pronounced increase in control samples (0 mM La DM) within 4 h. Treatment with 20 mM La DM did not alter H3K4me3 levels within 4 h, but after 24 h H3K4me3 was not detectable. **D** Representative pictures of C2C12 cells after 15 min of incubation with 0 or 20 mM La DM followed by IF staining for H3K4me3 (green) and DAPI (blue). **E** Quantification of H3K4me3 staining intensity in C2C12 cells showed decreased levels already after 15 min in 20 mM La DM conditions compared to 0mM treated cells. * indicates statistical significance, i.e. p < 0.05.

C2C12 myoblasts were incubated with 20 mM La DM for up to 24 hours and results are shown in Fig 1. Western blot analysis of lysed samples showed increased p-p38 after 30 min and 1 h of incubation in 0 mM La myoblasts but not in the 20 mM La treated samples (Fig 1B). H3K4me3 increased in control samples after 30 min with a considerable reduction after 24 hours. In contrast, La-treated myoblasts showed decreased H3K4me3 over time which was not detectable any more after 24 h (Fig 1C). Determination of nuclear H3K4me3 staining intensity by IF quantification showed an increase in H3K4me3 after 15 min in control but not La-treated samples (p = 0.002; Fig 1D + 1E). Staining intensity in control samples were not statistically different compared to 20 mM La myoblasts.

**Fig 2:**
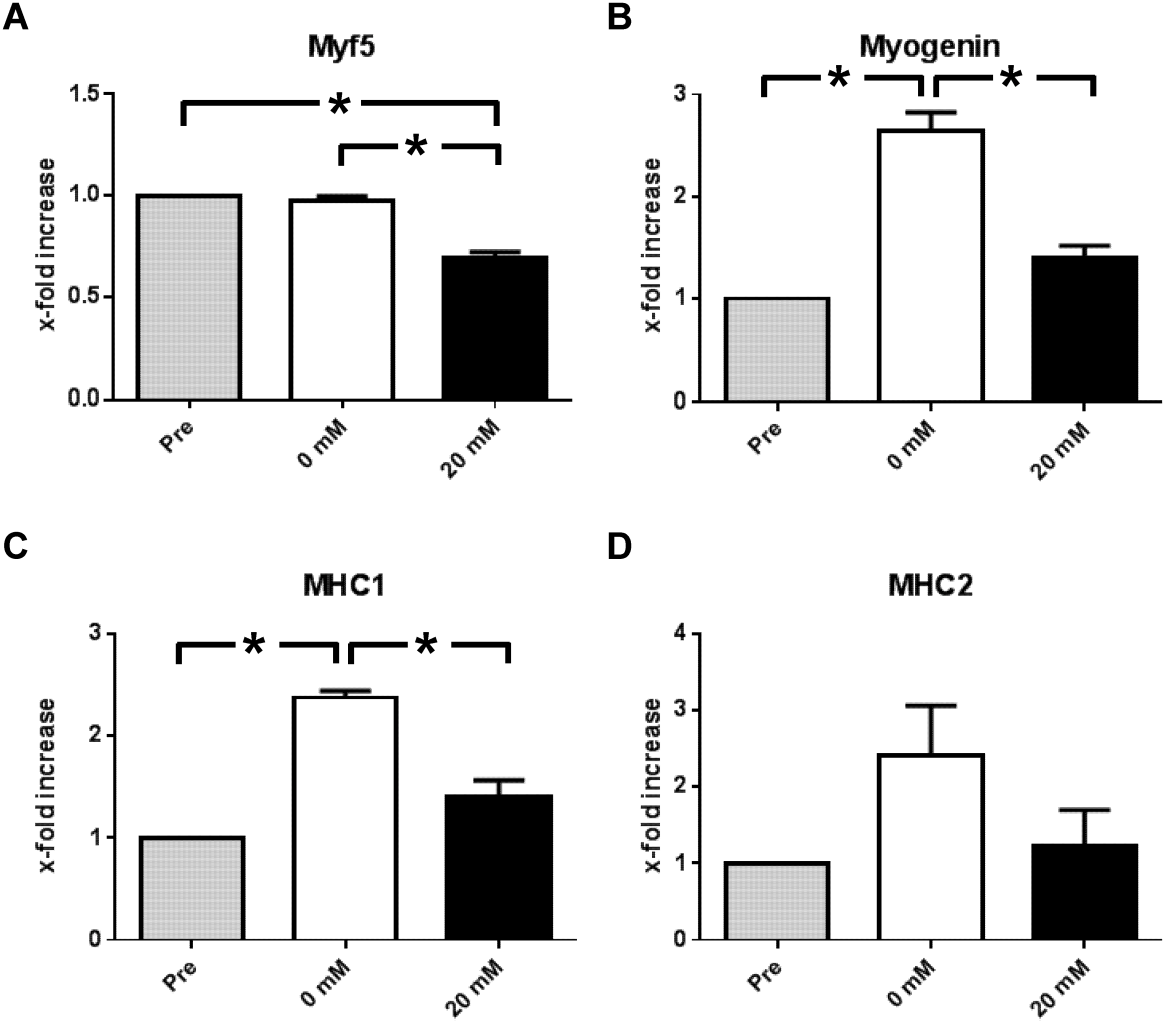
Acute effects of 2 h of 20 mM La DM incubation on muscle-specific gene expression in differentiating C2C12 myoblasts. **A** Expression of Myf5 did not change under control conditions but 20 mM La DM reduced Myf5 expression significantly. **B** Expression of myogenin was induced by control DM but 20 mM La inhibited an increase. **C** MHC1 expression was also induced by control DM. This effect was abolished by the addition of 20 mM La. **D** MHC2 expression remained unaffected by 20 mM La treatment. * indicates statistical significance, i.e. p < 0.05.

**Fig 3:**
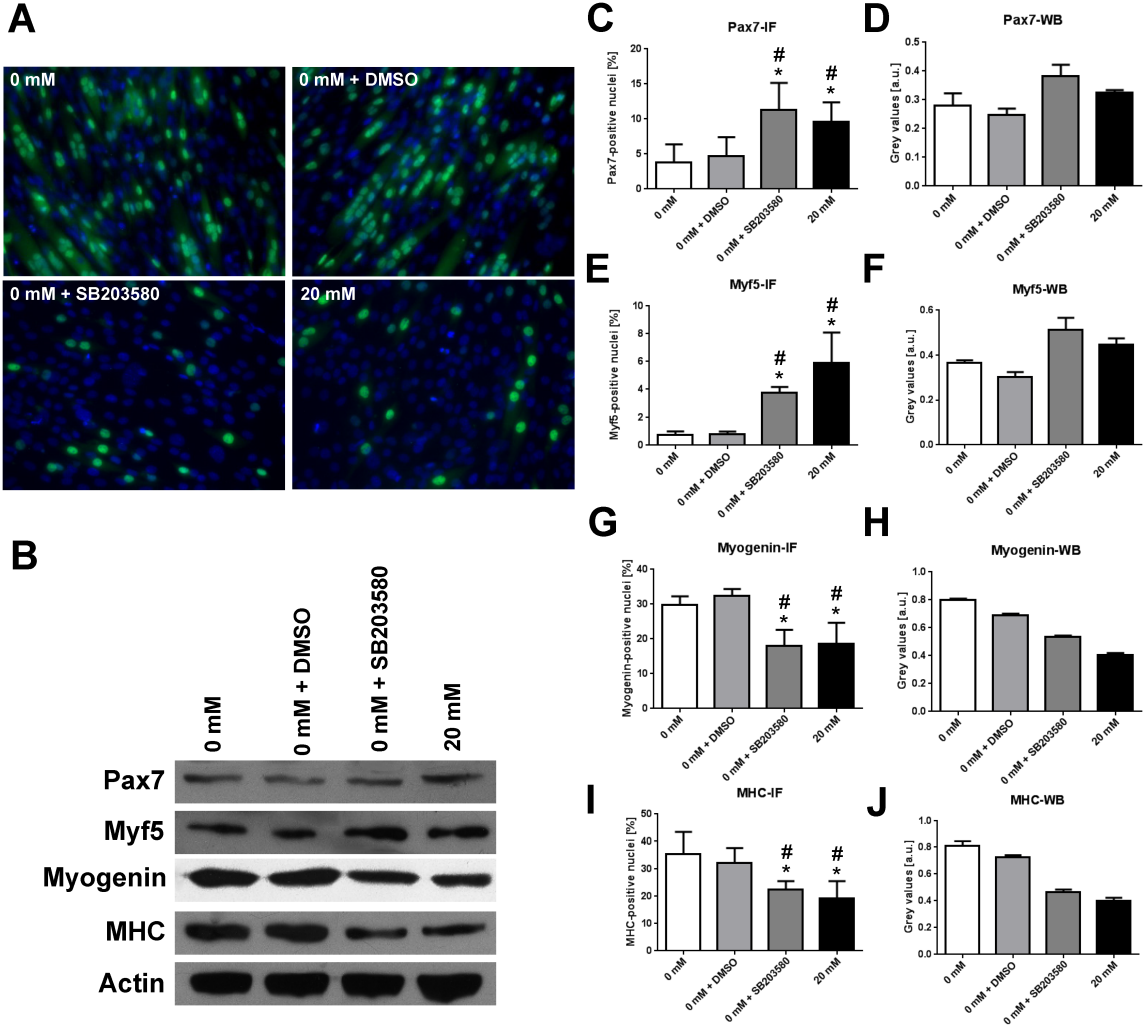
La-induced delay in late differentiation in C2C12 cells is similar to p38 MAPK-inhibition in cells treated with 0 mM La DM + SB203580. **A** Myogenin- (green, Alexa 488) and DAPI-staining (blue) in C2C12 myoblasts before (pre) and after 5 days of incubation with the respective DM. **B** Representative western blots for Pax7, Myf5, myogenin, and MHC. Actin was used as a loading control. **C, E, G, I** Ratio of positively stained nuclei in IF analysis. **D, F, H, J** Quantification of western blots for Pax7 (**C, D**), Myf5 (**E, F**), myogenin (**G, H**), and MHC (**I, J**). Data presented as mean ± SD. * indicates statistical significance, p < 0.05. For every marker analysed, differences between 0 mM La DM or 0 mM La DM + DMSO and 0 mM La DM + SB203580 or 20 mM La DM were significant (p<0.01).

Myf5, myogenin and MHC are important muscle-specific genes regulated by H3K4me3 and crucial for differentiation progression of myoblasts. Gene expression analysis (Fig 2) showed significantly decreased mRNA levels for Myf5 following La incubation (pre vs. 20 mM: p = 0.01; 0 mM vs. 20 mM: p = 0.033; Fig 2A). Myogenin expression (pre vs. 0 mM: p = 0.016) marks the commitment to differentiation in myoblasts. La-treatment however abolishes the increase in myogenin mRNA (0 vs. 20 mM: p = 0.046; Fig 2B). Likewise, MHC 1 expression was induced by control DM (pre vs. 0 mM: p = 0.027) but not by 20 mM La DM (0 mM vs. 20 mM: p = 0.044; Fig 2C). MHC2 appeared to be affected, but differences were not statistically significant (Fig 2D). Taken together, observations from the experiments indicate a pronounced loss of DM-induced p-p38 and H3K4me3 in La-treated cells and subsequently decreased gene expression of p38 MAPK- and H3K4me3 regulated genes. La treatment led to diminished p-p38 and reduced H3K4me3 as described above. As these events are important for myogenic progression, long-term effects of La treatment should include reduced differentiation of C2C12 myoblasts. To confirm this, results from the specific experiments are shown in Fig 3 and also available as supplemental file. Transcription factors that are highly abundant during the proliferation and early differentiation phase, i.e. Pax7 and Myf5, were more abundant in La- or SB203580-treated samples. In contrast, myogenin and MHC which appear during end-terminal differentiation were markedly reduced in both IF (Fig 3A, 3C, 3E, 3G and 3I) and WB analysis (Fig 3B, 3D, 3F, 3H and 3J) compared to control conditions.

Cell culture results were confirmed in human skeletal muscle after STD and HIT. La was significantly higher and more than doubled in HIT compared to STD (p<0.05) up to 10 min post RE. Table 1 shows blood lactate levels before (pre) and at different time points post RE.

**Table 1:** Blood lactate levels in subjects of STD and HIT RE groups before and in the early time course after a single session of resistance exercise.

	Blood Lactate (mmol/L)						
RE modes	Baseline (pre)	0 min post	2 min post	4 min post	6 min post	8 min post	10 min post
**STD**	1.07 ± 0.02	3.30 ± 0.65	3.44 ± 0.63	3.23 ± 0.64	2.92 ± 0.62	2.59 ± 0.56	2.33 ± 0.52
**HIT**	0.85 ± 0.10	4.09 ± 0.65	5.92 ± 0.51	6.04 ± 0.58	5.89 ± 0.69	5.47 ± 0.71	4.83 ± 0.69
**STD vs. HIT (P-value)**^**a**^	0.77	0.32	***<0*.*05***	***<0*.*01***	***<0*.*01***	***<0*.*01***	***<0*.*05***

* indicates statistical significance between STD and HIT with corresponding p-values given in the bottom row.

Blood lactate levels in HIT and STD at rest as well as 0, 2, 4, 6, 8 and 10 min after resistance exercise (mmol/L blood).

**Fig 4:**
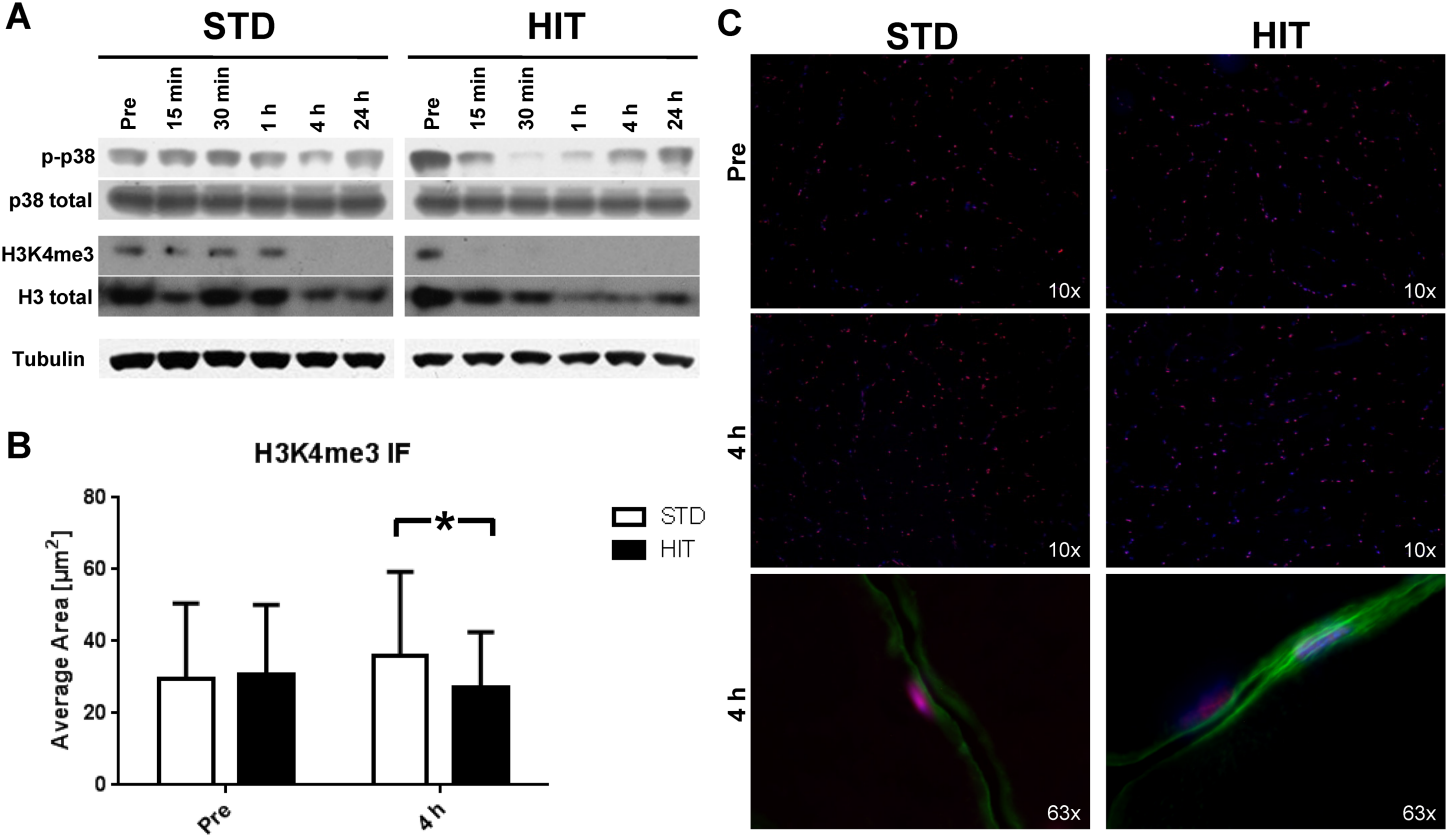
Acute effects of STD or HIT resistance exercise on p38 MAPK phosphorylation (p-p38) and H3K4 trimethylation (H3K4me3) *in vivo*. **A** Representative western Blots of human muscle homogenates at baseline (pre) and up to 24 h post RE. **B** Quantification of the average area of the H3K4me3 staining signal in human skeletal muscle nuclei pre and 4 h post STD and HIT demonstrating significantly lower nuclear H3K4me3 staining intensity following HIT compared to STD. **C** Representative pictures of human skeletal muscle cross-sections stained for H3K4me3 (red, Alexa 555), dystrophin as sarcolemma marker (green, Alexa 488) and DAPI (blue) before (pre) and after (4 h post) RE. Data presented as mean ± SD. * indicates statistical significance, i.e. p < 0.05.

Western blot analysis from muscle biopsy homogenates in STD showed unchanged phosphorylation of p38 at T180 and Y182 at 15, 30 min and 1 h post RE compared to baseline. This signal decreased after 4 h and before reaching baseline levels at 24 h post exercise. The HIT group displayed a reduction in p-p38 up to 30 min after the intervention with increasing levels at 1, 4, and 24 h post exercise (Fig 4A).

Next, whole muscle homogenates were analyzed for H3K4me3. In the STD group H3K4me3 content peaked 30 min post exercise but was not detectable at 4 and 24 h post exercise. In contrast, in the HIT group H3K4me3 was not detectable post the RE intervention (Fig 4A). Analysis of fixed muscle sections stained for H3K4me3 at baseline (pre) did not show any differences in the average area covered by the H3K4me3 signal between the two groups (Fig 4B + 4C). However, the average area significantly increased in the STD group (pre vs. 4 h post: p = 0.004), whereas the HIT group did not show changes from pre to 4 h post. Therefore, we determined a significantly lower H3K4me3 content at 4 h post-exercise following HIT compared to STD (p < 0.001). Fig 4C shows H3K4me3 stained myonuclei within skeletal muscle cross-sections of STD and HIT subjects revealing significantly higher H3K4me3 staining in myonuclei of STD than in HIT.

## Discussion

Lactate has been proposed as a metabolic signal for altering gene expression [4, 5, 20, 28] and therefore may contribute as regulator of myogenic adaptation following exercise. As the underlying mechanisms are largely unknown, this study demonstrates that incubating the cellular environment with 20mM La reduces p38 MAPK activation and H3K4me3 associated with a reduced expression of muscle specific genes in C2C12 myoblasts compared to 0mM lactate.

The novel finding of reduced p-p38 MAPK in C2C12 myoblasts following 20 mM La incubation is in line with the observation of decreased H3K4me3 and reduced muscle-specific gene expression. p38 MAPK directly phosphorylates Mef2d [12] leading to the activation of the HMT complex Ash2L, which establishes the H3K4me3 mark. This tight relationship was reflected in the timely similar activation pattern shown in Fig 1B + 1C. Further support of the hypothesis that La modulates gene expression via inhibition of p38 MAPK is supported by reduced gene expression of Myf5 and myogenin. Following 20 mM La DM incubation, gene expression levels of Myf5, myogenin and MHC1 were significantly decreased (Fig 2A–2C). As p-p38 and H3K4me3 are strong pro-myogenic regulators of muscle-specific gene expression, it was not surprising that repeated La-exposition led to reduced differentiation similar to the application of the specific p38 MAPK inhibitor SB203580 (Fig 3A).

During early stages of myoblast differentiation an increase in p-p38 MAPK can be observed (for a review, see [6]). To our knowledge, to date no study has specifically investigated the activation of p38 MAPK or H3K4me3 in response to La-treatment in differentiating myoblasts. One study found elevated p-p38 in adult rat ventricular myocytes as response to incubation with 20 mM La medium in order to simulate ischemia [15]. However, the ischemic incubation media used in this study contained sodium dithionite, a strong reducing agent (PubChem CID: 24489) that might account for the observed differences in p-p38 by the induction of oxidative stress. However, their results are in accordance with another study that used a very different experimental setup. Milanova and co-workers found increased p-p38 levels in vasculogenic stem/progenitor cells grown *in vivo* in La-supplemented matrigel plugs in mice [20]. In contrast, Mendler et al. demonstrated that La inhibits p-p38 and JNK activation in cytotoxic T cells (CTL), leading to CTL inhibition by tumor lactic acidosis [29]. Hence, the effects of La-induced regulation of p38 MAPK appear partly inconsistent and to depend on the disposed cell type and treatment protocol.

To test whether this pathway remains sensitive in human skeletal muscle upon exercise-induced stress, we applied STD and HIT RE on human subjects to acutely and locally induce increased metabolic demand and La-accumulation in skeletal muscle. For this purpose we chose single-leg eccentric and concentric HIT RE until maximum fatigue as a model of increased metabolic stress as it is accompanied by very high and locally elevated La-levels and requires also the recruitment of the majority of abundant myofibres [3, 21, 23, 30–35]. Although we were not able to determine intramuscular La levels, significantly higher systemic La-levels in HIT vs. STD were detected following single legged RE contractions from 4 min to 10 min after resistance exercise (Table 1). La-levels increased significantly in each RE mode (2–10 min post RE, p<0.01). Already at 4 min post RE, La-levels of around 6 mM (Table 1) doubled up to 10 min post RE compared to STD. This reflects La-levels usually observed during high intense endurance exercise [34, 36] or isokinetic strength exercise [35, 37] and dependent on a high local lactate accumulation. After 30 s of supramaximal exercise on a cycling ergometer, maximal intramuscular La concentrations in vastus lateralis were shown to be 73 mM*kg dry weight and higher [38] and hence up to 10-fold higher than in resting muscle and also several fold higher as determined in blood [39]. Considering the fact that in the current study the leg extensors of only one leg contributed to the changes in systemic La levels, we assume intramuscular La levels in the vastus lateralis muscle of our subjects to be significantly elevated and similar to that observed after high intense cycling exercise. As we used single leg exercise with a short exercise time (time under tension: ~70 s for both, HIT and STD) eliciting a generally lower systemic aerobic response [3, 24] than cycling exercise, we assume the metabolic rate accelerating the removal of La to be relatively low in the present study and maintaining elevated La levels for a sustained time course after HIT. In single cases we observed elevated La levels ~ 3 mM in HIT subjects 25 min post RE while La levels in STD were around baseline levels (~ 1.5 mM) within that time course (data not shown). We found decreasing p-p38 in HIT 30 min post RE but unchanged levels in STD assuming higher La-levels to be at least partially responsible for this effect. Interestingly some studies reported unchanged p-p38 immediately after RE [40] but a reduction of p-p38 within 2 and up to 5 hours after resistance exercise [40, 41] which corresponds to our observation. In contrast, some studies also reported increased p-p38 1 h after mechanically demanding and pure eccentric RE with only 6 repetitions and 5 min resting time [42] between sets. Although these authors did not determine La levels, a much lower La production compared to the HIT protocol in our study can be hypothesized, as eccentric exercise is less metabolically demanding than concentric exercise [30, 43, 44] and only few repetitions will not require maximum glycolytic activation [45]. The observed differences might thus be explained by the diversity of exercise regimens used in human exercise studies but also the timing of skeletal muscle biopsies. As we collected the first biopsy at 15 min post RE, an immediate post exercise-induced increase in p-p38 might have been missed and the observed reduction in p-p38 might occur as a time-dependent and delayed response.

Few studies have been published (reviewed in [46]) that describe exercise-induced histone modifications in skeletal muscle associated with the regulation of promoter regions of muscle specific genes. We added a tight time frame of post RE muscle biopsies to be able to assess temporal relationships between p-p38 and H3K4me3 histone modifications. To our knowledge, this is the first study that shows a link between metabolic stimulation by La and reduced p38 MAPK phosphorylation as well as lower H3K4me3 content and reduced myogenic gene expression in differentiating C2C12 cells. Due to limited sample size we were not able to carry out qRT-PCR analysis in the human subjects. However, it was demonstrated that in human skeletal muscle exercise-induced epigenetic modifications and subsequently altered gene expression depend on metabolic signals, e.g. AMPK and CaMKII [47]. Phosphorylation and activation of these kinases lead to nuclear export of histone deacetylases 4 and 5 (HDAC4 and 5) rendering these enzymes to suppress histone acetylation [46], a modification that is associated with transcriptional elongation. Another study demonstrated a reduction of DNA methylation of the promoters of PGC1α, PDK4, and PPAR-δ and increased gene expression in dependency of exercise intensity [48]. We determined that La as metabolic stimulus modulates cell signaling and epigenetic modifications via reduced p-p38 and H3K4me3, associated with changes in muscle-specific gene expression and phenotypical changes *in vitro*. Although we cannot exclude mechanical stimulation induced by RE to exert additional effects on p38 MAPK signaling [49] in adult skeletal muscle, our *in vitro* data indicate that the acute effects in skeletal muscle following HIT vs. STD is to a high extent metabolically driven. Further studies, focusing on detailed gene expression analysis and chromatin immunoprecipitation will be of importance, firstly to determine genes labelled with the H3K4me3 mark and secondly to elucidate the functional relevance of a transient suppression of myogenic gene expression.

As high intensity exercise is generally associated with beneficial results of skeletal muscle adaptation [26, 50], it has to be particularly determined whether acute metabolically demanding exercise offers detrimental or advantageous effects for myoblast differentiation and skeletal muscle adaptation under conditions of recurring metabolic stress. This will be of importance for the evaluation of high exercise intensity in training regimen under time efficient guidelines for competing athletes and in clinical settings. We have recently shown that especially eccentric contractions, likely associated with decreased metabolic demand [44] and La-production, offer higher anabolic responses compared to HIT and STD [26]. But although HIT RE showed potentially the lowest anabolic signaling response in this study, there were still significant increases in the acute responses towards adaptation and comparable to STD. Importantly, athletes that apply metabolic stress due to highly increased lactate production on a regular basis (e.g. sprinters) do not show impaired skeletal muscle adaptation. Hence, the observed time-dependent response of p38 and H3k4me3 in the current study might not blunt adaptability per se but rather emphasize a mechanism that transiently reduces signaling in order to temporarily prioritize the signaling network to fine tune directed adaptation and gene-expression.

## Supporting information

S1 Table La-induced differentiation delay in C2C12 cells is comparable to p38 MAPK-inhibition after 5 days of differentiation. (A) Mean percentages ± S.D. of C2C12 cells positive for the differentiation markers Pax7, Myf5, myogenin, and MHC. (B-E) P-values for the comparison of treatment groups (0 mM, 0 mM + DMSO, 0 mM + SB203580, 20 mM) for Pax 7 (B), Myf 5 (C), myogenin (D), and MHC (E).
(XLS)

## References

[1] Coffey VG, Hawley JA. The molecular bases of training adaptation. Sports Medicine. 2007;37(9):737–63. 17722947 10.2165/00007256-200737090-00001

[2] Fitts RH. Cellular mechanisms of muscle fatigue. Physiol Rev. 1994;74(1):49–94. 8295935 10.1152/physrev.1994.74.1.49

[3] Buitrago S, Wirtz N, Yue Z, Kleinöder H, Mester J. Mechanical load and physiological responses of four different resistance training methods in bench press exercise. J Strength Cond Res. 2013;27(4):1091–100. 10.1519/JSC.0b013e318260ec77 22692106

[4] Willkomm L, Schubert S, Jung R, Elsen M, Borde J, Gehlert S, et al Lactate regulates myogenesis in C2C12 myoblasts in vitro. Stem Cell Res. 2014;12(3):742–53. 10.1016/j.scr.2014.03.004 24735950

[5] Hashimoto T, Hussien R, Oommen S, Gohil K, Brooks GA. Lactate sensitive transcription factor network in L6 cells: activation of MCT1 and mitochondrial biogenesis. FASEB J. 2007;21(10):2602–12. 10.1096/fj.07-8174com 17395833

[6] Keren A, Tamir Y, Bengal E. The p38 MAPK signaling pathway: a major regulator of skeletal muscle development. Mol Cell Endocrinol. 2006;252(1–2):224–30. 10.1016/j.mce.2006.03.017 16644098

[7] Lluís F, Perdiguero E, Nebreda AR, Muñoz-Cánoves P. Regulation of skeletal muscle gene expression by p38 MAP kinases. Trends Cell Biol. 2006;16(1):36–44. 10.1016/j.tcb.2005.11.002 16325404

[8] de la Serna IL, Carlson KA, Imbalzano AN. Mammalian SWI/SNF complexes promote MyoD-mediated muscle differentiation. Nat Genet. 2001;27(2):187–90. 10.1038/84826 11175787

[9] de la Serna IL, Ohkawa Y, Berkes CA, Bergstrom DA, Dacwag CS, Tapscott SJ, et al MyoD targets chromatin remodeling complexes to the myogenin locus prior to forming a stable DNA-bound complex. Mol Cell Biol. 2005;25(10):3997–4009. 10.1128/MCB.25.10.3997-4009.2005 15870273 PMC1087700

[10] Simone C, Forcales SV, Hill DA, Imbalzano AN, Latella L, Puri PL. p38 pathway targets SWI-SNF chromatin-remodeling complex to muscle-specific loci. Nat Genet. 2004;36(7):738–43. 10.1038/ng1378 15208625

[11] Lluís F, Ballestar E, Suelves M, Esteller M, Muñoz-Cánoves P. E47 phosphorylation by p38 MAPK promotes MyoD/E47 association and muscle-specific gene transcription. EMBO J. 2005;24(5):974–84. 10.1038/sj.emboj.7600528 15719023 PMC554117

[12] Rampalli S, Li L, Mak E, Ge K, Brand M, Tapscott SJ, et al p38 MAPK signaling regulates recruitment of Ash2L-containing methyltransferase complexes to specific genes during differentiation. Nat Struct Mol Biol. 2007;14(12):1150–6. 18026121 10.1038/nsmb1316 PMC4152845

[13] Cole F, Zhang W, Geyra A, Kang JS, Krauss RS. Positive regulation of myogenic bHLH factors and skeletal muscle development by the cell surface receptor CDO. Dev Cell. 2004;7(6):843–54. 15572127 10.1016/j.devcel.2004.10.009

[14] Takaesu G, Kang JS, Bae GU, Yi MJ, Lee CM, Reddy EP, et al Activation of p38alpha/beta MAPK in myogenesis via binding of the scaffold protein JLP to the cell surface protein Cdo. J Cell Biol. 2006;175(3):383–8. 10.1083/jcb.200608031 17074887 PMC2064516

[15] Hawke TJ, Garry DJ. Myogenic satellite cells: physiology to molecular biology. J Appl Physiol (1985). 2001;91(2):534–51. 11457764 10.1152/jappl.2001.91.2.534

[16] Kadi F, Thornell LE. Concomitant increases in myonuclear and satellite cell content in female trapezius muscle following strength training. Histochem Cell Biol. 2000;113(2):99–103. 10766262 10.1007/s004180050012

[17] Petrella JK, Kim JS, Cross JM, Kosek DJ, Bamman MM. Efficacy of myonuclear addition may explain differential myofiber growth among resistance-trained young and older men and women. Am J Physiol Endocrinol Metab. 2006;291(5):E937–46. 10.1152/ajpendo.00190.2006 16772322

[18] McKinnell IW, Ishibashi J, Le Grand F, Punch VG, Addicks GC, Greenblatt JF, et al Pax7 activates myogenic genes by recruitment of a histone methyltransferase complex. Nat Cell Biol. 2008;10(1):77–84. 10.1038/ncb1671 18066051 PMC2739814

[19] Aziz A, Liu QC, Dilworth FJ. Regulating a master regulator: establishing tissue-specific gene expression in skeletal muscle. Epigenetics. 2010;5(8):691–5. 10.4161/epi.5.8.13045 20716948 PMC3052885

[20] Milovanova TN, Bhopale VM, Sorokina EM, Moore JS, Hunt TK, Hauer-Jensen M, et al Lactate stimulates vasculogenic stem cells via the thioredoxin system and engages an autocrine activation loop involving hypoxia-inducible factor 1. Mol Cell Biol. 2008;28(20):6248–61. 10.1128/MCB.00795-08 18710947 PMC2577432

[21] Hunter GR, Seelhorst D, Snyder S. Comparison of metabolic and heart rate responses to super slow vs. traditional resistance training. J Strength Cond Res. 2003;17(1):76–81. 12580660 10.1519/1533-4287(2003)017<0076:comahr>2.0.co;2

[22] Lagally KM, Robertson RJ, Gallagher KI, Goss FL, Jakicic JM, Lephart SM, et al Perceived exertion, electromyography, and blood lactate during acute bouts of resistance exercise. Med Sci Sports Exerc. 2002;34(3):552–9; discussion 60. 11880823 10.1097/00005768-200203000-00025

[23] Szivak TK, Hooper DR, Dunn-Lewis C, Comstock BA, Kupchak BR, Apicella JM, et al Adrenal cortical responses to high-intensity, short rest, resistance exercise in men and women. J Strength Cond Res. 2013;27(3):748–60. 10.1519/JSC.0b013e318259e009 22561973

[24] Thornton MK, Potteiger JA. Effects of resistance exercise bouts of different intensities but equal work on EPOC. Med Sci Sports Exerc. 2002;34(4):715–22. 11932584 10.1097/00005768-200204000-00024

[25] Kyriakis JM, Avruch J. Mammalian MAPK signal transduction pathways activated by stress and inflammation: a 10-year update. Physiol Rev. 2012;92(2):689–737. 10.1152/physrev.00028.2011 22535895

[26] Gehlert S, Suhr F, Gutsche K, Willkomm L, Kern J, Jacko D, et al High force development augments skeletal muscle signalling in resistance exercise modes equalized for time under tension. Pflugers Arch. 2014. 10.1007/s00424-014-1579-y 25070178

[27] Pfaffl MW. A new mathematical model for relative quantification in real-time RT-PCR. Nucleic Acids Res. 2001;29(9):e45 11328886 10.1093/nar/29.9.e45 PMC55695

[28] Hashimoto T, Brooks GA. Mitochondrial lactate oxidation complex and an adaptive role for lactate production. Med Sci Sports Exerc. 2008;40(3):486–94. 10.1249/MSS.0b013e31815fcb04 18379211

[29] Mendler AN, Hu B, Prinz PU, Kreutz M, Gottfried E, Noessner E. Tumor lactic acidosis suppresses CTL function by inhibition of p38 and JNK/c-Jun activation. Int J Cancer. 2012;131(3):633–40. 10.1002/ijc.26410 21898391

[30] Beltman JGM, de Haan A, Haan H, Gerrits HL, van Mechelen W, Sargeant AJ. Metabolically assessed muscle fibre recruitment in brief isometric contractions at different intensities. European Journal of Applied Physiology. 2004;92(4–5):485–92. 10.1007/s00421-004-1105-6 15138833

[31] Altenburg TM, Degens H, van Mechelen W, Sargeant AJ, de Haan A. Recruitment of single muscle fibers during submaximal cycling exercise. Journal of Applied Physiology. 2007;103:1752–6. 10.1152/japplphysiol.00496.2007 17823300

[32] Godin R, Ascah A, Daussin FN. Intensity-dependent activation of intracellular signalling pathways in skeletal muscle: role of fibre type recruitment during exercise. J Physiol. 2010;588(Pt 21):4073–4. 10.1113/jphysiol.2010.195925 21037317 PMC3002441

[33] Nardone A, Schieppati M. Shift of activity from slow to fast muscle during voluntary lengthening contractions of the triceps surae muscles in humans. J Physiol. 1988;395:363–81. 3411483 10.1113/jphysiol.1988.sp016924 PMC1191999

[34] Jacobs I, Bar-Or O, Karlsson J, Dotan R, Tesch P, Kaiser P, et al Changes in muscle metabolites in females with 30-s exhaustive exercise. Med Sci Sports Exerc. 1982;14(6):457–60. 7162392 10.1249/00005768-198206000-00009

[35] Jacobs I. Lactate concentrations after short, maximal exercise at various glycogen levels. Acta Physiol Scand. 1981;111(4):465–9. 10.1111/j.1748-1716.1981.tb06764.x 7304208

[36] Bergman BC, Wolfel EE, Butterfield GE, Lopaschuk GD, Casazza GA, Horning MA, et al Active muscle and whole body lactate kinetics after endurance training in men. J Appl Physiol (1985). 1999;87(5):1684–96. 10562610 10.1152/jappl.1999.87.5.1684

[37] Tesch P. Muscle fatigue in man. With special reference to lactate accumulation during short term intense exercise. Acta Physiol Scand Suppl. 1980;480:1–40. 6933824

[38] Jacobs I, Tesch PA, Bar-Or O, Karlsson J, Dotan R. Lactate in human skeletal muscle after 10 and 30 s of supramaximal exercise. J Appl Physiol Respir Environ Exerc Physiol. 1983;55(2):365–7. 6618929 10.1152/jappl.1983.55.2.365

[39] Karlsson J. Lactate and phosphagen concentrations in working muscle of man with special reference to oxygen deficit at the onset of work. Acta Physiol Scand Suppl. 1971;358:1–72. 5549478

[40] Møller AB, Vendelbo MH, Rahbek SK, Clasen BF, Schjerling P, Vissing K, et al Resistance exercise, but not endurance exercise, induces IKKβ phosphorylation in human skeletal muscle of training-accustomed individuals. Pflugers Arch. 2013;465(12):1785–95. 10.1007/s00424-013-1318-9 23838844

[41] Widegren U, Jiang XJ, Krook A, Chibalin AV, Björnholm M, Tally M, et al Divergent effects of exercise on metabolic and mitogenic signaling pathways in human skeletal muscle. FASEB J. 1998;12(13):1379–89. 9761781 10.1096/fasebj.12.13.1379

[42] Tannerstedt J, Apro W, Blomstrand E. Maximal lengthening contractions induce different signaling responses in the type I and type II fibers of human skeletal muscle. Journal of Applied Physiology. 2009;106(4):1412–8. 10.1152/japplphysiol.91243.2008 19112158

[43] Krustrup P, Soderlund K, Mohr M, Gonzalez-Alonso J, Bangsbo J. Recruitment of fibre types and quadriceps muscle portions during repeated, intense knee-extensor exercise in humans. Pflugers Archiv-European Journal of Physiology. 2004;449(1):56–65. 10.1007/s00424-004-1304-3 15290298

[44] Bigland-Ritchie B, Woods JJ. Integrated electromyogram and oxygen uptake during positive and negative work. J Physiol. 1976;260(2):267–77. 978517 10.1113/jphysiol.1976.sp011515 PMC1309091

[45] Baker JS, McCormick MC, Robergs RA. Interaction among Skeletal Muscle Metabolic Energy Systems during Intense Exercise. J Nutr Metab. 2010;2010:905612 10.1155/2010/905612 21188163 PMC3005844

[46] McGee SL, Hargreaves M. Histone modifications and exercise adaptations. J Appl Physiol (1985). 2011;110(1):258–63. 21030677 10.1152/japplphysiol.00979.2010

[47] McGee SL, Fairlie E, Garnham AP, Hargreaves M. Exercise-induced histone modifications in human skeletal muscle. J Physiol. 2009;587(Pt 24):5951–8. 10.1113/jphysiol.2009.181065 19884317 PMC2808551

[48] Barrès R, Yan J, Egan B, Treebak JT, Rasmussen M, Fritz T, et al Acute exercise remodels promoter methylation in human skeletal muscle. Cell Metab. 2012;15(3):405–11. 10.1016/j.cmet.2012.01.001 22405075

[49] Martineau LC, Gardiner PF. Insight into skeletal muscle mechanotransduction: MAPK activation is quantitatively related to tension. J Appl Physiol. 2001;91(2):693–702. 11457783 10.1152/jappl.2001.91.2.693

[50] Gibala M. Molecular responses to high-intensity interval exercise. Appl Physiol Nutr Metab. 2009;34(3):428–32. 10.1139/H09-046 19448710

